# Optimized Pinecone-Squama-Structure MoS_2_-Coated CNT and Graphene Framework as Binder-Free Anode for Li-Ion Battery with High Capacity and Cycling Stability

**DOI:** 10.3390/ma16083218

**Published:** 2023-04-19

**Authors:** Hanwen Jian, Tongyu Wang, Kaiming Deng, Ang Li, Zikun Liang, Erjun Kan, Bo Ouyang

**Affiliations:** MLLT Key Laboratory of Semiconductor Microstructure and Quantum Sensing, Department of Applied Physics, Nanjing University of Science and Technology, Nanjing 210094, China; 317113010165@njust.edu.cn (H.J.); liang2100@njust.edu.cn (A.L.); ekan@njust.edu.cn (E.K.)

**Keywords:** molybdenum sulfide, carbon nanotubes-graphene, optimized structure, 3D framework architecture, lithium-ion battery

## Abstract

Extensive research has been conducted on the development of high-rate and cyclic stability anodes for lithium batteries (LIBs) due to their high energy density. Molybdenum disulfide (MoS_2_) with layered structure has garnered significant interest due to its exceptional theoretic Li^+^ storage behavior as anodes (670 mA h g^−1^). However, achieving a high rate and long cyclic life of anode materials remains a challenge. Herein, we designed and synthesized a free-standing carbon nanotubes-graphene (CGF) foam, then presented a facile strategy to fabricate the MoS_2_-coated CGF self-assembly anodes with different MoS_2_ distributions. Such binder-free electrode possesses the advantages of both MoS_2_ and graphene-based materials. Through rational regulation of the ratio of MoS_2_, the MoS_2_-coated CGF with uniformly distributed MoS_2_ exhibits a nano pinecone-squama-like structure that can accommodate the large volume change during the cycle process, thereby significantly enhancing the cycling stability (417 mA h g^−1^ after 1000 cycles), ideal rate performance, and high pseudocapacitive behavior (with a 76.6% contribution at 1 mV s^−1^). Such a neat nano-pinecone structure can effectively coordinate MoS_2_ and carbon framework, providing valuable insights for the construction of advanced anode materials.

## 1. Introduction

The rapid growth of portable electronic devices, electric vehicles, and grid energy technologies has created a significant challenge in energy storage due to the increasing demands of modern civilization [1,2,3,4]. Rechargeable lithium-ion batteries (LIBs) have emerged as one of the most significant energy storage devices due to their high energy density and low environmental impact [5,6]. In a continuous effort by the research community to develop high-performance rechargeable batteries, electrode materials that follow alternative mechanisms have been investigated, such as alloying anodes and transition metal sulfides. However, alloying anode-based batteries suffer from the large volumetric expansion of anodes and associated phenomena during battery cycling [7].

Benefiting from the two-dimensional layered structure, MoS_2_ comprises sandwiched S–Mo–S layers with an interlayer spacing of ~6.7 Å, which allows Li-ion insertion between layers, similar to graphite [8]. MoS_2_ has been regarded as a promising anode candidate, which enables a high theoretical capacity of 670 mA h g^−1^ [9,10]. However, MoS_2_ anodes suffer from low electrical conductivity and electrode deterioration during cycling; after reactions with Li^+^-ions, MoS_2_ electrodes are enriched with polysulfide species (as reaction products) and partially dissolve in the battery electrolyte [11,12], which leads to low rate capability and rapid capacity degradation [8,13]. Low electron conductivity is particularly problematic with the use of standard conductive additives (e.g., carbon particles ∼50–200 nm in diameter), which tend to lose electrical contact with the active particles during the conversion reactions. Great efforts have been devoted to overcoming these restrictions, including reducing particle size to alleviate strain [14], hybridizing MoS_2_ with conductive materials such as graphene [15,16,17,18,19,20], carbon nanotubes (CNTs) [21,22,23], and carbon polymers [24,25,26,27].

Most current studies concentrated on compositing MoS_2_ with various morphologies of carbon materials, which has addressed the problem of MoS_2_ electrode deterioration by reducing the quantity of MoS_2_. Typically, these are ultrathin MoS_2_ nano-sheets supported on N-doped carbon nanoboxes and hierarchical MoS_2_ tubular structures wired by carbon nanotubes; both nanocomposites have provided excellent lithium-ion storage behaviors [28,29]. However, these electrodes are largely dependent on a complicated fabrication process along with the binder introduction during cell assembly, which inevitably increase the electrode expense. Additionally, the complex process can hardly control the uniform distribution of MoS_2_ on carbon materials, which results in rapid agglomeration of active materials during cycling, which is the primary cause of MoS_2_ electrode deterioration.

In this study, we present a facile approach for the fabrication of a pinecone-squama-like MoS_2_ nano-sheet coated on carbon nanotube–graphene–foam (s-MoS_2_@CGF) electrode. The CGF framework serves as the substrate for MoS_2_ growth, providing adequate conductivity and structural strength. Moreover, the interconnected 3D hierarchical structure offers a favorable surface area for MoS_2_ loading, facilitating charge transfer and accommodating the strain release during cycling, reducing the formation of the gel-like polymeric layer from S dissolution in electrolyte [29,30,31,32]. As a self-supported electrode, the as-prepared s-MoS_2_@CGF anode exhibits the original performance of MoS_2_ and CGF while avoiding the effect of binders and conductive additives. The pinecone-squama-like MoS_2_ uniformly loaded on the CGF surface through intermolecular force and C-S bond helps to prevent MoS_2_ aggregation and effectively accommodates the volume changes in MoS_2_ [33]. Additionally, the nano-sized MoS_2_ coating on the CGF surface shortens the Li^+^ diffusion distance, enhances electron transport behavior, and provides high Li^+^ storage performance [28]. To investigate the impact of MoS_2_ distribution on electrode performance, we also synthesized a nano-flower morphology MoS_2_ sample (f-MoS_2_@CGF). The distribution of MoS_2_ turned into non-uniform and agglomerated to a nano-flower morphology along with the increase in MoS_2_ nano-sheets. Despite the increased loading amount of MoS_2_, the performance of the f-MoS_2_@CGF electrode is not as good as the s-MoS_2_@CGF electrode, which has a uniform distribution of MoS_2_ on the CGF substrate. This is due to the lack of close connection between the un-uniformed MoS_2_ nano-sheets and the carbon backbone. As a result, the unguided MoS_2_ nano-sheets tend to agglomerate and deteriorate the anode performance during cycling resulting in bad performance.

## 2. Materials and Methods

### 2.1. Growth of CGF Film

The CGF was grown via the typical chemical vapor deposition (CVD) approach. Initially, a piece of Ni foam (NF) was subjected to several rounds of cleaning using deionized water and ethanol. Next, the NF was immersed in an ethanol solution comprising 10 wt.% ethylene glycol and 0.1 M Ni(NO_3_)_2_ for 1 min and then dried at 75 °C for 1 h. The dried NF was placed into the center of a quartz tube. Under a gas flow consisting of H_2_ (5%) and Ar (95%), the quartz tube was heated to 600 °C and remained for 30 min with ethanol placed in a gas wash bottle and introduced by gas flow as the carbon source. Subsequently, the furnace was rapidly cooled down to room temperature. The free-standing CGF could be obtained after etching the Ni template via 1 M FeCl_3_ solution. The typical areal mass of obtained CGF film was ~1.0 mg cm^−2^.

### 2.2. Synthesis of s-MoS_2_@CGF and MoS_2_ Powders

The initial MoS_2_ and MoS_2_ anchored conductive graphene foam (s-MoS_2_@CGF and f-MoS_2_@CGF) were prepared through a hydrothermal method. In brief, a precursor solution was prepared by dissolving 60 mg ammonium molybdate (Sinopharm Chemical Reagent Co., Ltd., Shanghai, China) and 80 mg thiourea (Macklin) in 50 mL deionized water with ultrasonication. After the above materials were completely dissolved, one piece of CGF film was immersed in the precursor solution and then transferred into a Teflon-lined stainless autoclave. Then, the autoclave was sealed, and a hydrothermal reaction was carried out at 180 °C for 12 h. Following cooling to room temperature, the sample was rinsed multiple times with DI water and dried at 60 °C for 3 h in an oven. The obtained sample was then annealed at 350 °C for 3 h under a mixed gas flow consisting of 5% H_2_ and 95% Ar at a heating rate of 5 °C min^−1^. The areal mass of s-MoS_2_@CGF was approximately 2.3 mg cm^−2^. For comparison, MoS_2_ powders were synthesized similarly without the introduction of CGF.

### 2.3. Synthesis of f-MoS_2_@CGF

The f-MoS_2_@CGF was synthesized in the same way as s-MoS_2_@CGF by adjusting the amount of Mo and S and had a mass of around 2.9 mg cm^−2^. The precursor solution was prepared by dissolving 90 mg ammonium molybdate (Sinopharm Chemical Reagent Co., Ltd.) and 120 mg thiourea (Macklin) in 50 mL deionized water with ultrasonication, and the rest remained the same.

### 2.4. Characterization

The X-ray diffraction (XRD) results were collected by a Bruker-AXS D8 Advance diffractometer with Cu_Kα_ line (λ = 1.5406 Å). Raman spectra were obtained with the Jobin Yvon LabRAM Aramis system with a 532 nm excitation laser at room temperature. The X-ray photoelectron spectroscope (XPS) measurements were performed with the PHI QUANTERA II system using a monochromatic Al_Kα1_ (1486.6 eV) as an X-ray source. The morphology characterizations of all samples were carried out by JSM-IT500HR scanning electron microscope (SEM) and JEOL-2100F transmission electron microscope (TEM).

### 2.5. Electrochemical Measurements

The anode performance of all synthesized materials was evaluated by assembling coin-type cells CR 2032 in an argon-filled glove box with oxygen and moisture contents less than 0.1 ppm. All prepared materials were directly used as electrodes without introducing copper foil and binding additives. Metallic lithium foil was used as a counter and reference electrode, and 1 M LiPF_6_ in ethylene carbonate (EC)–diethylene carbonate (DEC) (V/V = 1:1) was used as the electrolyte. A polypropylene (PP) film (Cellgard 2400) was used as the separator. The anode material had a mass of approximately 2.2–2.5 mg cm^−2^, and the size of self-supported materials was 0.5 × 0.5 cm^2^. Galvanostatic charge–discharge (GCD) tests were performed with different current rates using a NEWARE battery resting apparatus. Cyclic voltammetry (CV) measurements were conducted using the bio-logic electrochemical workstation, and electrochemical impedance spectroscopy (EIS) was carried out over a frequency range from 0.1 to 10^6^ Hz after 10 cycles of the galvanostatic charge–discharge (GCD) test.

## 3. Results

**Figure 1:**
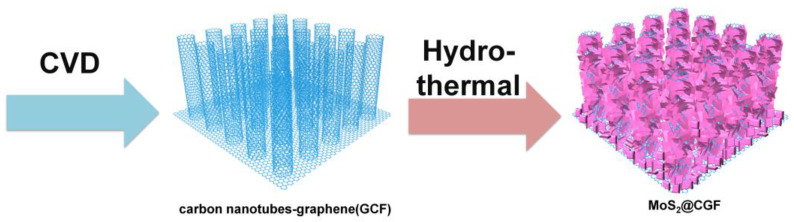
Schematic illustration of the synthesis process of MoS_2_@CGF.

**Figure 2:**
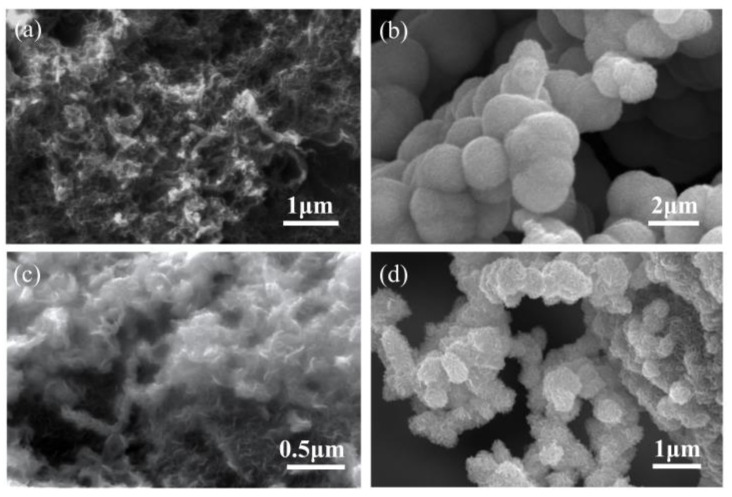
SEM image of (**a**) CGF, (**b**) bare MoS2, (**c**) s-MoS2@CGF, and (**d**) f-MoS2@CGF.

The flexible MoS_2_@CGF electrode was synthesized through two simple processes illustrated in Figure 1, and it demonstrated excellent capacity and cycling performance. The profile of the 3D free-standing CGF (Figure S1) exhibits an interconnected macro-porous structure. As shown in Figure 2a, numerous cross-linked CNTs were directly grown on GF, which resulted in increased active sites for MoS_2_. In terms of bare MoS_2,_ as shown in Figure 2b, the achieved nano-sheets were aggregated towards nano-flower-like structures with a radius of ~1.5 μm. When the carbon-based substrate was introduced (Figure 2c), hierarchical MoS_2_ nano-sheets uniformly covered the CGF surface, forming a pinecone-squama-like nanostructure, which suggests the protective effect of CNTs and graphene network on the growth of MoS_2_ from aggregation. As the amount of MoS_2_ increased, the nano-sheets aggregated into a nano-flower structure and exhibited a random distribution on the surface of MoS_2_@CGF (Figure 2d), leading to the deterioration of the MoS_2_@CGF anode [10].

**Figure 3:**
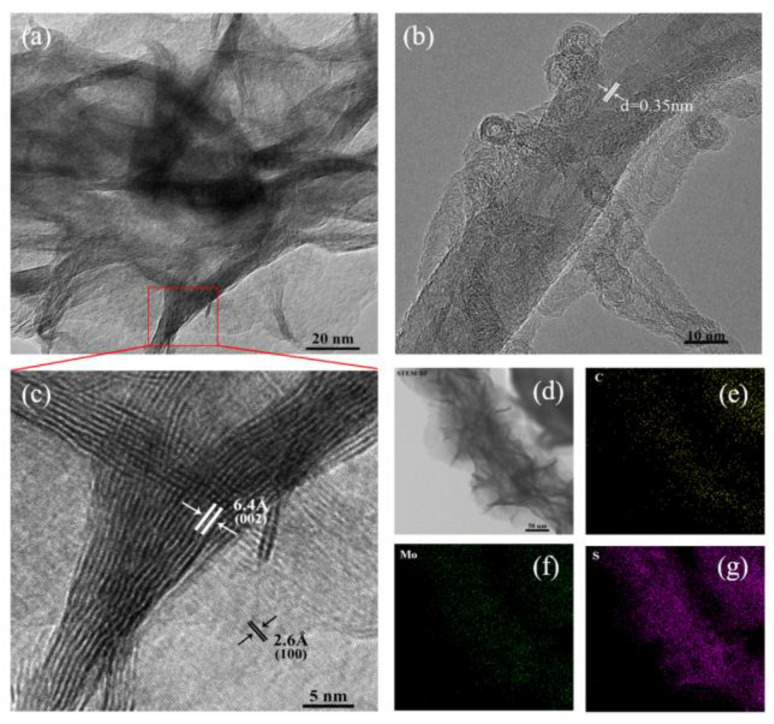
TEM image of (**a**) CGF; (**b**) s-MoS2@CGF; (**c**) TEM image of the s-MoS2@CGF; (**d**) STEM image of s-MoS2@CGF; elemental mapping images of (**e**) C, (**f**) Mo, and (**g**) S.

TEM images in Figure 3 reveal the detailed structure of CGFs and s-MoS_2_@CGF. Figure 3a shows the CNTs with an interplanar distance of ~0.35 nm, which is consistent with the (002) planes of CNTs. As depicted in Figure 3b, MoS_2_ was grown on the surface of hierarchically oriented CNTs. Figure 3c displays the typical layered crystal structure of MoS_2_ with a (002) plane of CNTs. As depicted in Figure 3b, MoS_2_ was grown on the surface of hierarchically oriented CNTs. Figure 3c shows the typical layered crystal structure of MoS_2_ with a lattice spacing of 0.64 nm, consistent with the (002) plane of hexagonal MoS_2_, and a lattice spacing of 0.26 nm, corresponding to the (100) plane. In addition, Figure 3d presents the elemental distribution of s-MoS_2_@CGF studied by energy dispersive spectroscopy (EDS) mapping, demonstrating that the MoS_2_ squama is perpendicularly grown on the CNTs’ backbone.

**Figure 4:**
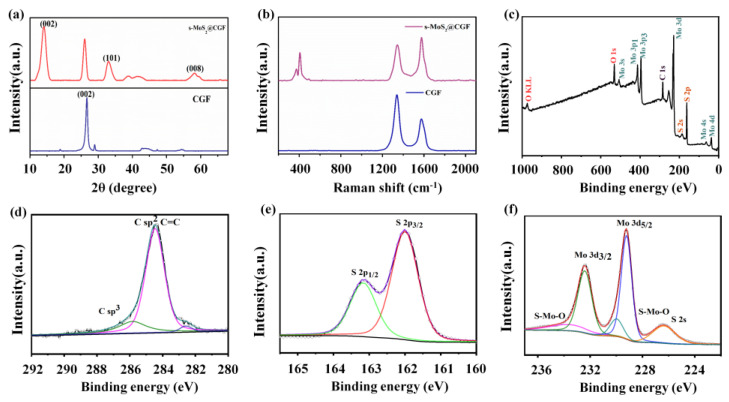
(**a**) XRD pattern; (**b**) Raman spectra of the prepared CGF and MoS_2_@CGF composite; (**c**) total XPS spectrum of MoS_2_@CGF; XPS spectra of MoS_2_@CGF in (**d**) C 1s, (**e**) S 2p, and (**f**) Mo 3d, respectively.

The X-ray diffraction (XRD) patterns of both CGF and s-MoS_2_@CGF exhibit a well-defined and strong peak at 26.5° in Figure 4a, which corresponds to the (002) plane of graphitic carbon (JCPDS card No. 65-6212). This peak indicates that the CGF film has a highly crystalline graphitic structure. Moreover, the diffraction peaks observed in s-MoS_2_@CGF at 14°, 32°, and 59° can be attributed to the (002), (100), (103), and (110) planes of MoS_2_ (JCPDS card no. 37-1492) [34,35,36]. Raman spectroscopy was utilized to further investigate the microstructure of CGF and s-MoS_2_@CGF (Figure S2 and Figure 4b). Two characteristic peaks at 380 and 405 cm^−1^ are associated with the E^1^_2g_ and A_1g_ vibration modes of MoS_2_. E^1^_2g_ mode is mainly caused by the interlayer displacement of S and Mo, and A_1g_ mode is attributed to out-layer symmetric displacements of S. Two strong peaks at ~1340 and ~1580 cm^−1^ can be attributed to D-band and G-band, respectively. According to the CGF sample, the ratio of I_D_/I_G_ is 1.69, demonstrating a significant amount of active sites for Li^+^ storage [16,28,37]. The I_D_/I_G_ decreases to 1.15 for s-MoS_2_@CGF, indicating that numerous defects were restored during MoS_2_ growth. The XRD and Raman spectra of f-MoS_2_@CGF are consistent with s-MoS_2_@CGF.

We employed X-ray photoelectron spectroscopy (XPS) to investigate the surface states, including components and chemical states, of s-MoS_2_@CGF, which were found to be similar to f-MoS_2_@CGF. The XPS full spectrum (Figure 4c) confirms the presence of Mo, S, C, and O elements. As shown in Figure 4d, the C 1s spectrum exhibits two peaks at 284.5 and 285.8 eV, which can be assigned to the sp^2^ carbon of CGF and sp^3^ carbon of C-C and C-S, respectively [33]. Notably, a tiny peak is located at 282.6 eV, which is attributed to the residual Ni after acid removal. The S 2p spectrum of MoS_2_@CGF shown in Figure 4e can be fitted by two-component peaks at 163.2 and 162.0 eV, which belongs to the S 2p_1/2_ and S 2p_3/2_ of S^2-^ in MoS_2_ [38]. The Mo 3d spectrum (Figure 4f) is divided into three peaks at 232.4, 229.2, and 226.3 eV corresponding to Mo^4+^ 3d_3/2_, Mo^4+^ 3d_5/2,_ and S 2s, respectively, which further confirms the successful growth of MoS_2_ [39,40]. Notably, the small peak at 235.1 eV is fitted to S-Mo-O caused by the oxidation of MoS_2_ [16].

**Figure 5:**
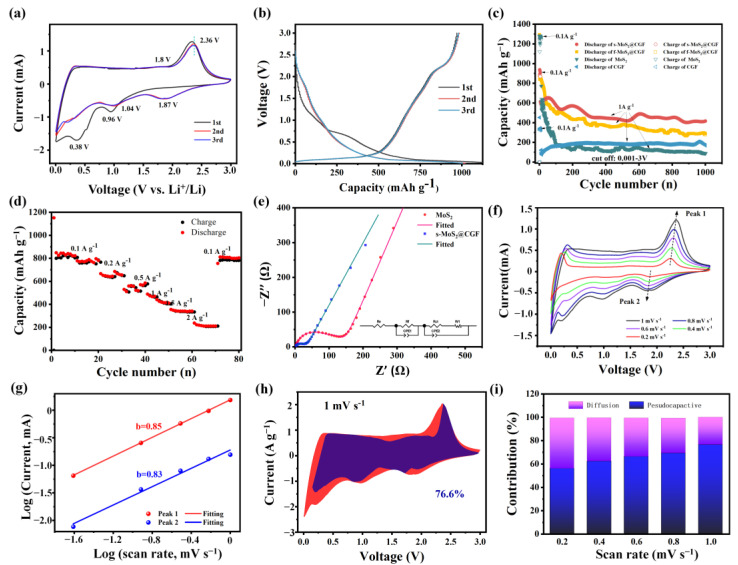
(**a**) CV curves of s-MoS_2_@CGF electrode in different cycles; (**b**) selected charge–discharge voltage profiles; (**c**) cycling performance; (**d**) rate capability; (**e**) EIS spectra e after 10 cycles recorded in the frequency range of 0.1–10^6^ Hz; (**f**) CV curves of s-MoS_2_@CGF at different scan rates; (**g**) logarithm peak current versus logarithm scan rate at peak 1 and peak 2; (**h**) Voltammetric responses for s-MoS_2_@CGF at sweep rate of 1mV s^−1^, the specific pseudocapacitive contribution is shown in purple region; (**i**) proportion of pseudocapacitive contribution at different scan rates.

The electrochemical characteristics of s-MoS_2_@CGF and f-MoS_2_@CGF were studied and compared with bare MoS_2_ and CGF. The initial three cycles of the s-MoS_2_@CGF electrode’s CV curves are presented in Figure 5a, which are comparable to the CV curves of f-MoS_2_@CGF (Figure S3). Two reduction peaks at 0.38 and 0.96 V were observed during the 1st discharging process. The reduction peak at 0.96 V can be attributed to the insertion of Li^+^ into MoS_2_ to create Li_x_MoS_2_ [8]. The peak at 0.38 V is associated with the reduction in Li_x_MoS_2_ to metallic Mo and Li_2_S, along with the formation of a solid electrolyte interface (SEI) layer [16]. The reaction can be represented as MoS_2_+4Li^+^+4e^−^→Mo+2Li_2_S [7]. During the anodic oxidation process, the weak oxidation peak at 1.8 V can be ascribed to the partial oxidation process from Mo to MoS_2_, while the subsequently pronounced peak at 2.34 V is associated with the oxidation of Li_2_S to S. Moreover, there is a new reduction peak at 1.87 V corresponding to the lithiation reaction of S to Li_2_S in the following cycles. The subsequent CV curves after the first cycle are retainable, indicating excellent structural stability of s-MoS_2_@CGF during electrochemical processes. However, compared with s-MoS_2_@CGF, the CV curves of the f-MoS_2_@CGF show a noticeable decline, confirming that the non-uniform distribution of MoS_2_ exacerbates the anode deterioration. The CV curves of bare CGF are presented in Figure S4, which is consistent with the previous reports of graphene-based materials [41]. In the case of bare MoS_2_ (Figure S5), the CV curves exhibit reduction peaks at 0.23 and 0.82 V and oxidation peak at 2.33 V during the first cycle [8,42], which vanished during the subsequent cycles, indicating the poor electrochemical performance of bare MoS_2_. Compared with the CV curves of the MoS_2_ anode, the oxidation peak of s-MoS_2_@CGF has a slight negative shift, and two reduction peaks have a positive shift (Figure S9), which could be caused by the interaction of the MoS_2_ and CGF, further supporting the strong combination of MoS_2_ and CGF [42].

Figure 5b shows the representative GCD profiles of s-MoS_2_@CGF at 0.1 A g^−1^. According to the CV curve, there are two voltage plateaus at around ~1.0 and ~0.5 V during the first discharge process. The potential plateau at ~1.0 V can be attributed to the formation of Li_x_MoS_2_, while the plateau at 0.5 V can be assigned to the conversion reaction of MoS_2_ to Mo and Li_2_S. Moreover, a distinct plateau between 0.1 and 0.5 V can only be observed in the first cycle, corresponding SEI formation along with Li^+^ intercalation into graphitic carbon [10]. A pronounced peak at around 2.3 V can be assigned to the delithiation of Li_2_S to S in the first charge process. In the following cycles, the potential plateaus become inconspicuous because of the nanocrystallization and amorphization during repeated charge and discharge processes, as shown in Figure S12 [29,33]. The initial discharging and charging capacities of the s-MoS_2_@CGF with 56.5% MoS_2_ electrode were 1192 and 969 mA h g^−1^, respectively. The Coulombic efficiency of the first and second discharge capacity is 81.9%, mainly resulting from the SEI formation [43]. The discharge profiles of the second and third cycles almost overlap, indicating the extraordinary stability of s-MoS_2_@CGF. In comparison, Figure S6 shows the initial discharge and charge capacity of f-MoS_2_@CGF (1212 and 992 mA h g^−1^), which is similar to s-MoS_2_@CGF. Additionally, the discharging capacity of f-MoS_2_@CGF with 65.5% MoS_2_ has a slight decrease in the second and third cycles, confirming that the non-uniform MoS_2_ could not solve the electrode deterioration problem. Moreover, the GCD performance of bare CGF and MoS_2_ were also investigated to realize the synergy of MoS_2_ and CGF in MoS_2_@CGF. As shown in Figure S7, the discharging capacity of CGF in first cycle is 344 mA h g^−1^, which is much lower than that of MoS_2_ and MoS_2_@CGF. Concerning the GCD performance of MoS_2_ (Figure S8), the initial capacity is 1064 mA h g^−1^ and has an obvious decrease in the next cycle, confirming that the combination of MoS_2_ and CGF can improve the stability of the MoS_2_@CGF electrode.

In Figure 5c, the cycling performance of various electrodes was assessed at a current density of 1 A g^−1^. Pinecone-squama-structure and nano-flower-structure MoS_2_@CGF electrodes both exhibited superior cycling stability compared to bare MoS_2_. The s-MoS_2_@CGF electrode demonstrated a slight decrease in capacity from 610 mA h g^−1^ to 451 mA h g^−1^ during the first 300 cycles due to the independent MoS_2_ and the non-uniform distribution of MoS_2_ on CGF (Figure S10). Then, the capacity remained stable in the following cycles, and after 1000 cycles, the capacity was about 417 mA h g^−1^ with a decay rate of 7.6%. There was an increase in capacity after ~550 cycles, and we believed that a partial electrode activation process occurred. Conversely, the electrode with non-uniform MoS_2_ distribution showed inferior cycling stability, with the f-MoS_2_@CGF capacity dropping from 850 to 310 mA h g^−1^ in 1000 cycles. Even though f-MoS_2_@CGF contains more MoS_2_ than s-MoS_2_@CGF, its long-term recyclable capacity is lower. Moreover, the bare MoS_2_ electrode showed a reversible capacity that rapidly reduced from 773 mA h g-1 to 160 mA h g^−1^ during the first 100 cycles. The capacity further degraded to 86 mA h g^−1^ after 1000 cycles, indicating a sharp electrode deterioration during the cycling. The capacity of CGF in the first 200 cycles slightly increased due to the activation of carbon materials and then stabilized at ~180 mA h g^−1^ in the subsequent 800 cycles. The excellent stability and high reversible capacity of both s-MoS_2_@CGF and f-MoS_2_@CGF can be attributed to the combination of 3D CGF foam and MoS_2_, which is further supported by the capacity performance of bare MoS_2_ and CGF electrode. Additionally, s-MoS_2_@CGF outperformed f-MoS_2_@CGF due to the uniform distribution of MoS_2_.

The present study also investigated the capacity rate of the s-MoS_2_@CGF hybrid at various current densities, and the results are shown in Figure 5d. The composite electrode displayed a good rate performance, with average specific capacities of 874.5, 821.7, 699.5, 580.6, 461.6, 361.5, and 223.8 mA h g^−1^ at the current densities of 0.1, 0.2, 0.5, 1, 1.5, 2, and 4 A g^−1^, respectively. Upon returning the current density to 0.1 A g^−1^, the capacity remained at 851.0 mA h g^−1^, which was slightly lower than the initial 10 cycles at 0.1 A g^−1^, indicating excellent reversibility of the s-MoS_2_@CGF electrode. This result suggests satisfactory structure stability and fast ion transfer during the cycling process, which is ascribed to the expanded space by CGF and the squama-structure of MoS_2_.

The electrochemical performances of s-MoS_2_@CGF for Li^+^ storage were further investigated utilizing EIS measurement in Figure 5e, providing valuable insights into the underlying mechanisms. The equivalent circuit was used with a modified Randle’s model, which contains a series resistance R_e_, charge transfer resistance R_ct_, and SEI-layer resistance Rf with a Warburg diffusion element W and constant-phase elements CPE1 and CPE2, as shown in the inset of Figure 5e. CPE1 corresponds to capacitance to SEI film, and CPE2 is the electrical double layer (EDL) capacitance of the electrode/electrolyte interface. Inhomogeneities in the surface of metal oxide electrodes result in nonideal capacitance in the double layer at the solid/electrolyte interface. For this reason, CPEs are routinely used in place of pure capacitors to model this interfacial layer [44]. The value of CPE1 and CPE2 of MoS_2_ are 3.5 × 10^−6^ and 8.7 × 10^−5^ F·cm^−2^·s^α−1^), and the value of CPE1 and CPE2 of s-MoS_2_@CGF are 4.6 × 10^−6^ and 3.9 × 10^−5^ F·cm^−2^·s^α−1^. The Nyquist plots intersect with X-axis to reflect the resistance of the electrolyte Re, consisting of two semicircles at a high-frequency range, corresponding to the SEI layer’s resistance (R_f_) and the charge transfer resistance (R_ct_) at the interface of the electrode and electrolyte. The inclined line in the low-frequency region can be assigned with Warburg impedance (W), which is attributed to the diffusion of lithium in the bulk of the electrode. The value of Re, R_f,_ and R_ct_ of s-MoS_2_@CGF are 2.47, 21.74, and 9.98 Ω, respectively. In contrast, the Re, R_f,_ and R_ct_ of MoS_2_ are much higher than s-MoS_2_@CGF, which are 2.64, 81.8, and 59.04 Ω, respectively. These findings suggest that the electrical conductivity of s-MoS_2_@CGF is improved by utilizing carbon material as a framework, thus enhancing the electrochemical activity of MoS_2_ during cycling.

We calculated the pseudocapacitive contribution of s-MoS_2_@CGF from CV curves at different scan rates in Figure 5f to further study the relationship between lithium diffusion and capacitive charge storage in the present system. In general, there is a linear relationship between the peak currents (*i*) and scan rates (*v*) after the logarithm according to the following equations [25,45,46].

(1) $$i=av^{b},$$

(2) $$log\left(i\right)=blog\left(v\right)+log\left(a\right)\;$$

where *a* and *b* are variable parameters, through the linear relationship between logarithm current *log*(*i)* and logarithm scanning rate *log*(*v)*, the value of *b* can be calculated, which is the slope of *log*(*i)* and *log*(*v)*. The value of *b* can directly reflect the charge storage kinetics. The *b*-value of 0.5 represents a diffusion-controlled behavior, while the value of 1 indicates a standard capacitive performance. The values of *b* shown in Figure 5g are 0.85 and 0.83, corresponding to the cathodic peak and anodic peak, respectively, which illustrates the high pseudocapacitive behavior of such a free-standing electrode. Further, the pseudocapacitive performance can be directly determined by the equation:

(3) $$i\left(V\right)=k_{1}v+k_{2}v^{\frac{1}{2}}$$

where *k*_1_*v* represents the capacity effect, and $k_{2}v^{\frac{1}{2}}\;$ is on behalf of the diffusion-controlled behavior. In particular, the pseudocapacitive contribution of s-MoS_2_@CGF at 1 mV s^−1^ is approximately 76.6% (Figure 5h). Moreover, as shown in Figure 5i, the contribution of pseudocapacity is positively relevant to the scan rate. The result confirms that the pseudocapacitive Li^+^ storage is a majority in MoS_2_@CGF; this benefits the rating performance due to the fast electrochemical kinetics of pseudocapacitive Li^+^ storage.

## 4. Conclusions

In summary, we presented a facile approach for the synthesis of 3D hierarchical MoS_2_@CGF nanocomposites with various MoS_2_ distributions. The CGF backbone provides not only sufficient active sites for MoS_2_ growth but also provides ample space for the release of strain caused by the volume change in MoS_2_ during cycling. Moreover, the hierarchical nano-frameworks ensure the efficient interconnection of the entire anode, facilitating fast charge transport and reducing the diffusion length of Li^+^. MoS_2_ exhibits excellent battery performance, but the MoS_2_ distribution structure significantly affects the overall performance of MoS_2_@CGF. Non-uniform MoS_2_ distribution results in agglomeration into a nano-flower structure similar to bare MoS_2_, leading to electrode deterioration during cycling. However, uniform MoS_2_ distribution on carbon material forms a pinecone-squama structure that significantly improves anode stability during cycling, indicating the ability of this structure to accommodate the large volume changes in MoS_2_ and mitigate electrode degradation. As a binder-free electrode, s-MoS_2_@CGF demonstrates outstanding electrochemical performance, including high specific capacity, long cycling stability, excellent rate performance, and satisfactory pseudocapacitive performance. This study provides an effective strategy for constructing advanced LIB electrode materials by combining two complementary materials with an optimal structure.

## Data Availability

The data presented in this study are available in the article.

## Supplementary Materials

The following supporting information can be downloaded at: https://www.mdpi.com/article/10.3390/ma16083218/s1. References [47,48,49,50,51] are cited in the supplementary materials.
