# Supplementary material for: Optimized Pinecone-Squama-Structure MoS2-Coated CNT and Graphene Framework as Binder-Free Anode for Li-Ion Battery with High Capacity and Cycling Stability
Source: Materials (Basel). 2023 Apr 19;16(8):3218. doi: 10.3390/ma16083218 (PMC10143248; doi:10.3390/ma16083218)
Supplement: Supplementary file 1 [file materials-16-03218-s001.zip › materials-2318162-Supplementary.pdf]

Supplementary Information

Optimized Pinecone-Squama-Structure MoS_2-Coated CNT and Graphene Framework as Binder-Free Anode for Li-Ion Battery with High Capacity and Cycling Stability

Hanwen Jian †, Tongyu Wang *,†, Kaiming Deng, Ang Li, Zikun Liang, Erjun Kan and Bo Ouyang *

MLLT Key Laboratory of Semiconductor Microstructure and Quantum Sensing, Department of Applied Physics, Nanjing University of Science and Technology, Nanjing 210094, China

* Correspondence: tywang@njust.edu.cn (T.W.); ouyangboyi@njust.edu.cn (B.O.)

† These authors contributed equally to this work.

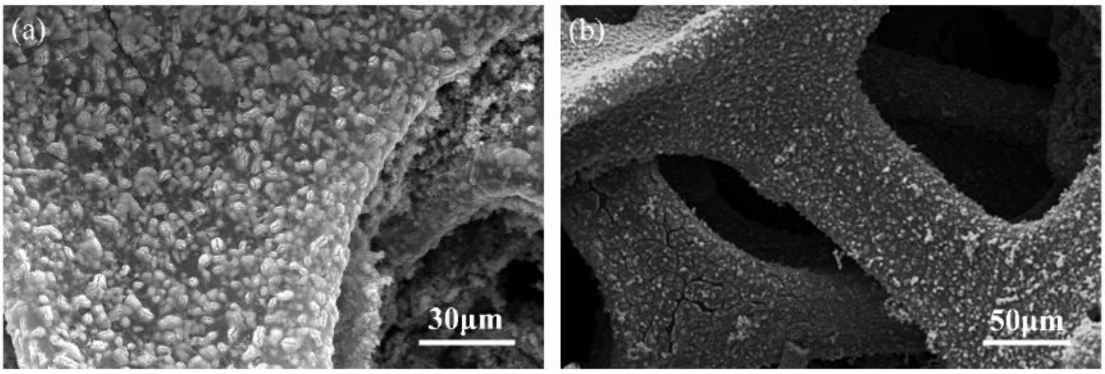

Figure S1. (a) CGF framework, (b) s-MoS_2@CGF framework.

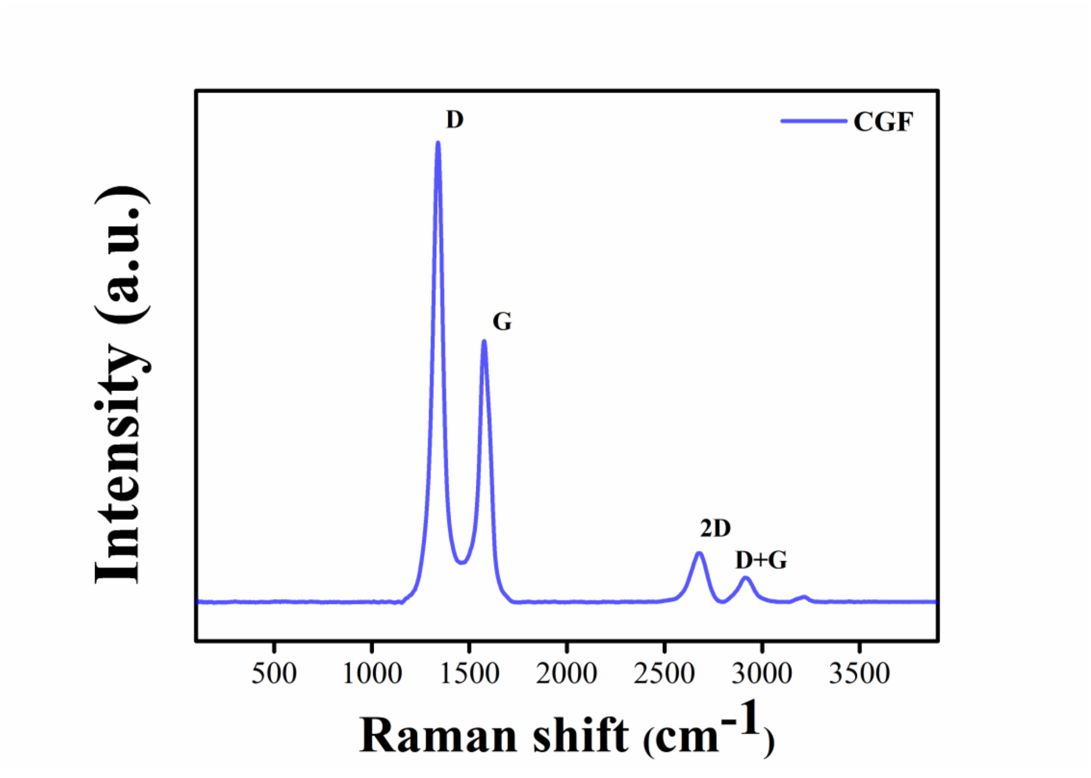

Figure S2. Raman spectrum of pure CGF.

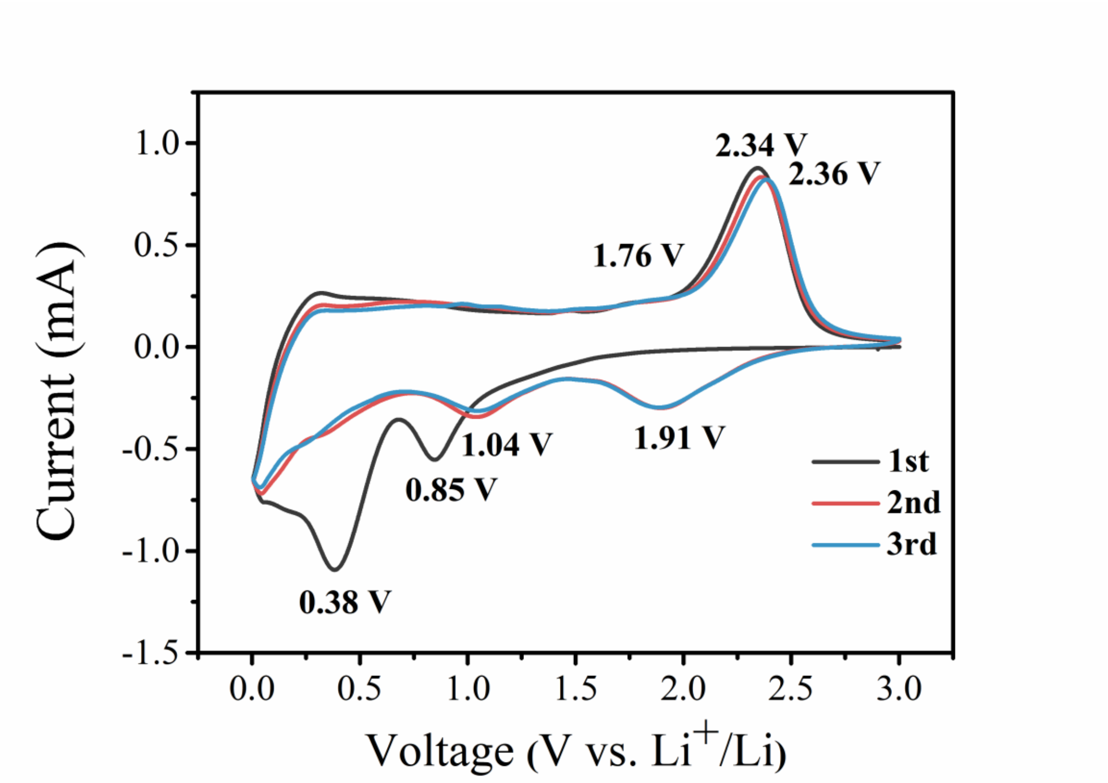

Figure S3. CV curves of f-MoS_2@CGF electrode at 1 mV/s in the different cycles.

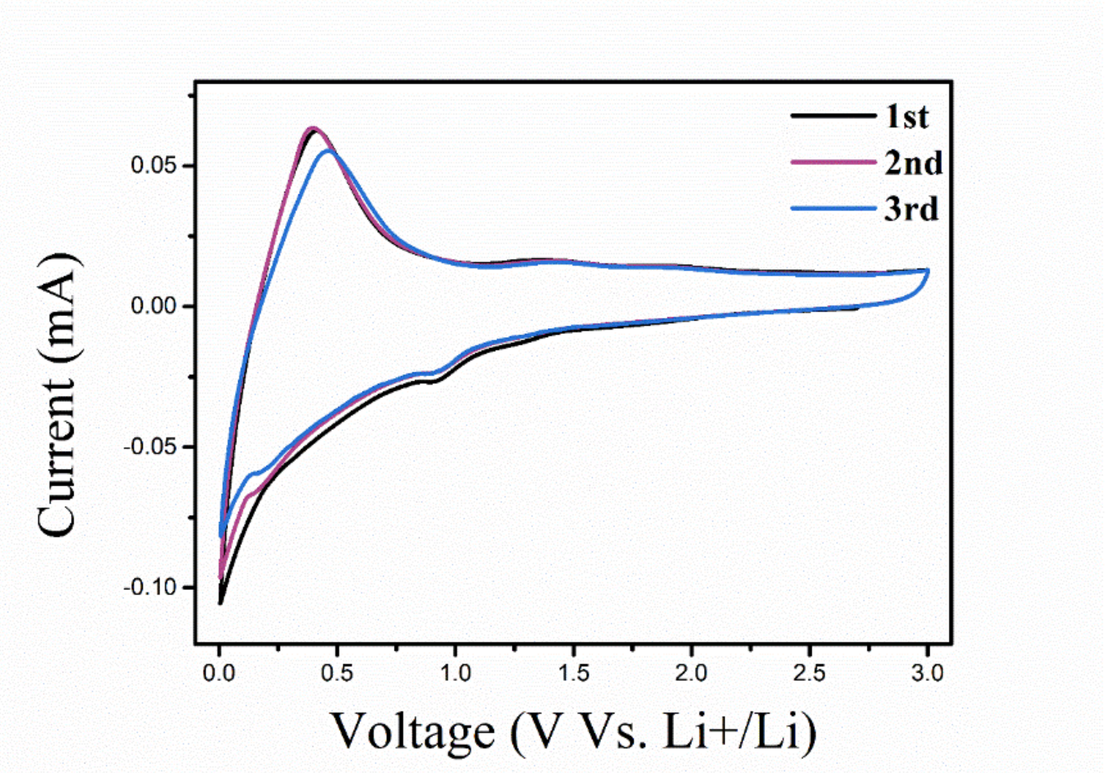

Figure S4. CV curves of bare CGF electrode at 1 mV/s in the different cycles.

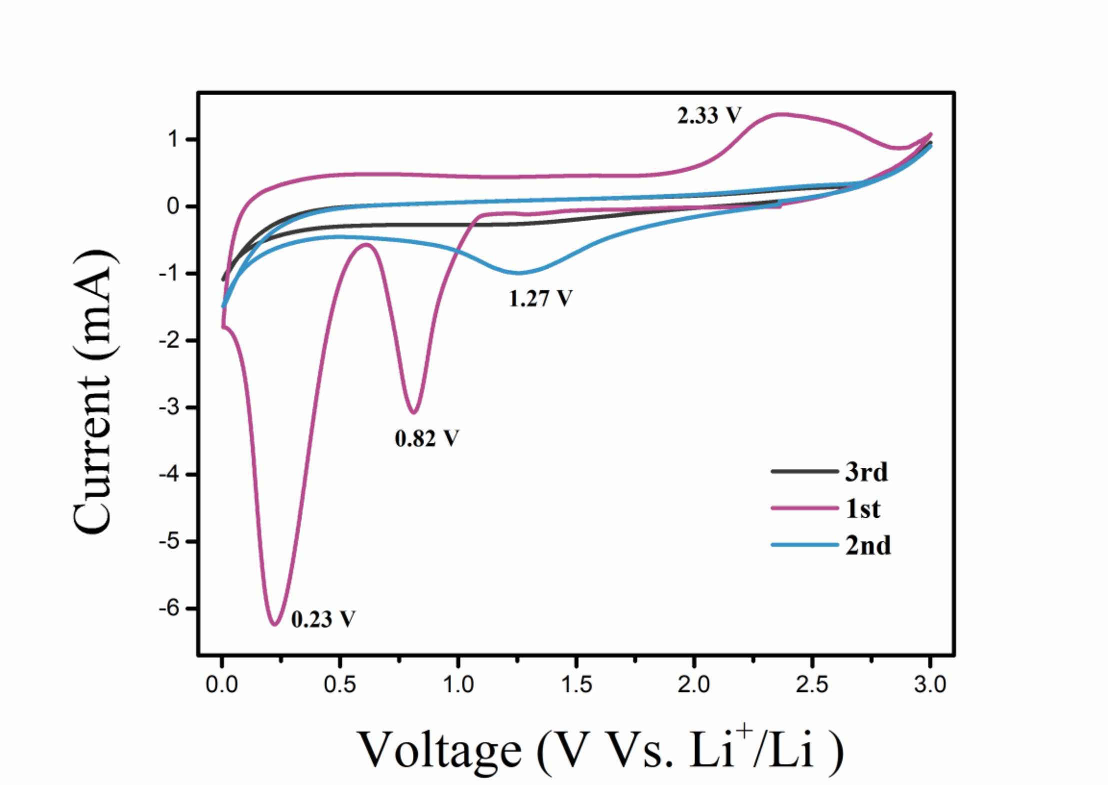

Figure S5. CV curves of bare MoS_2 electrode at 1 mV/s in the different cycles.

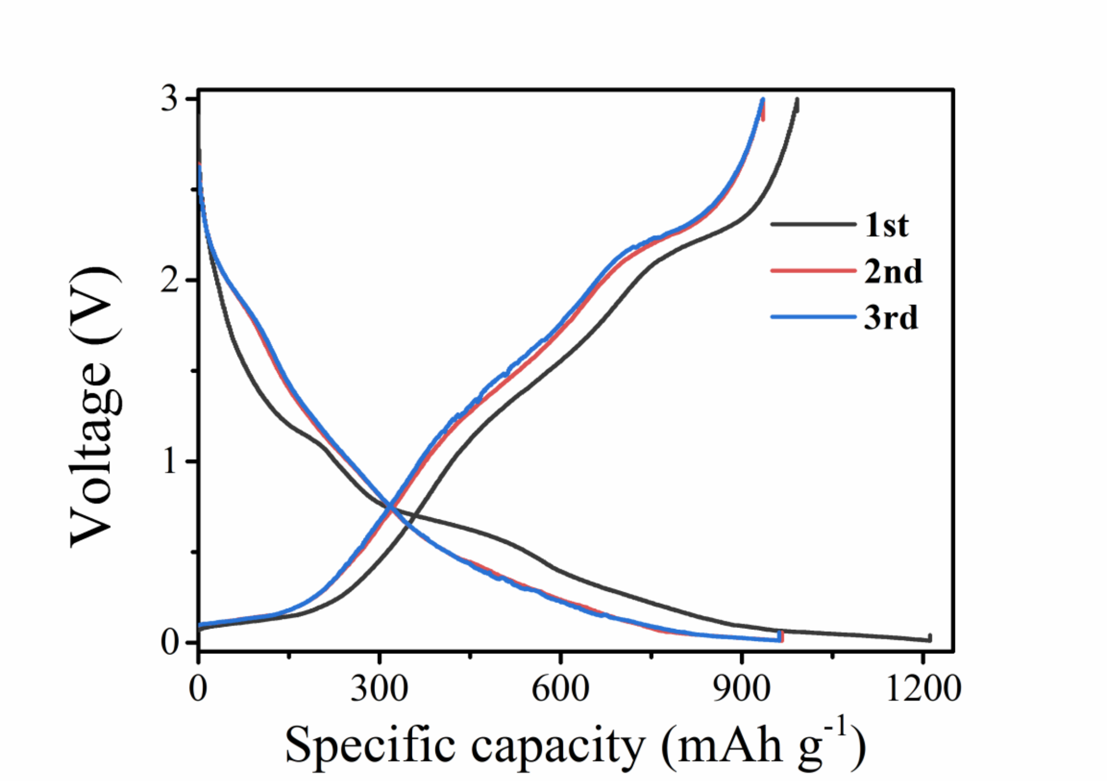

Figure S6. The charge-discharge profiles of f-MoS_2@CGF.

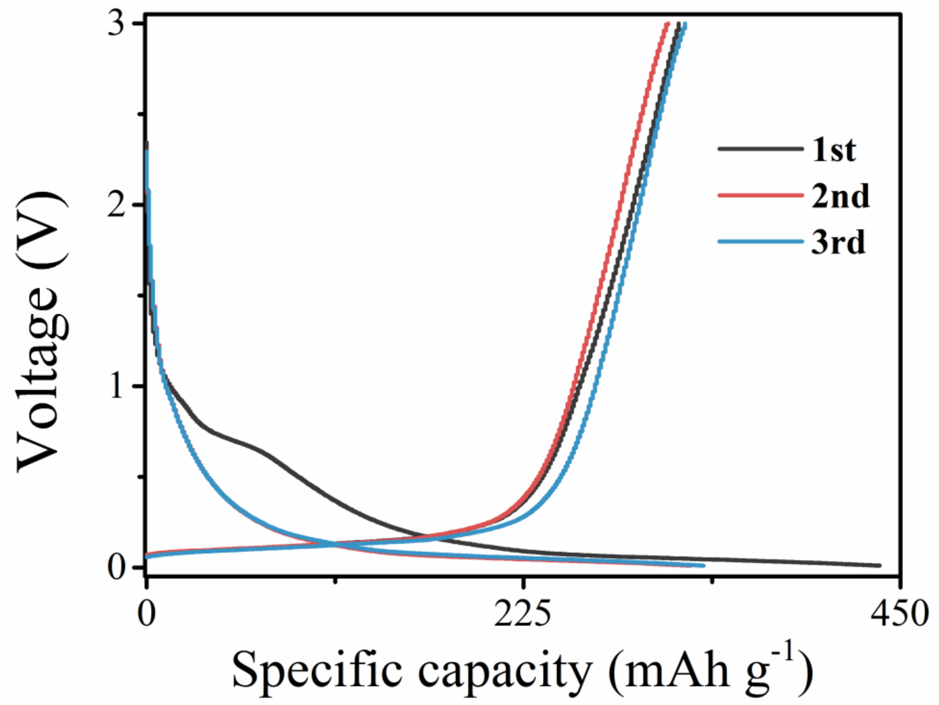

Figure S7. The charge-discharge profiles of CGF.

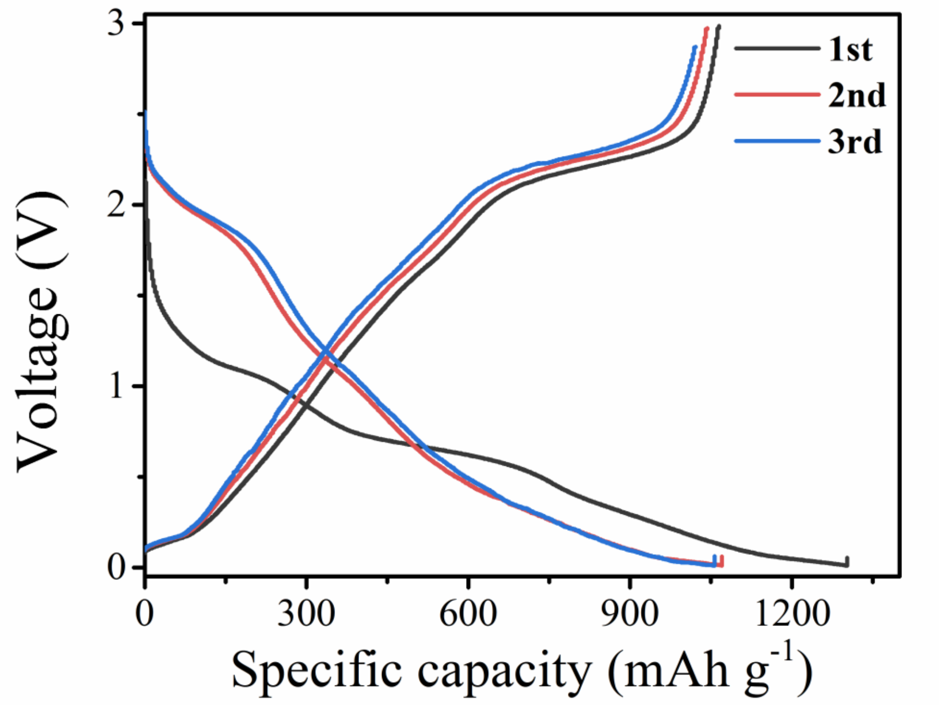

Figure S8. The charge-discharge profiles of MoS_2.

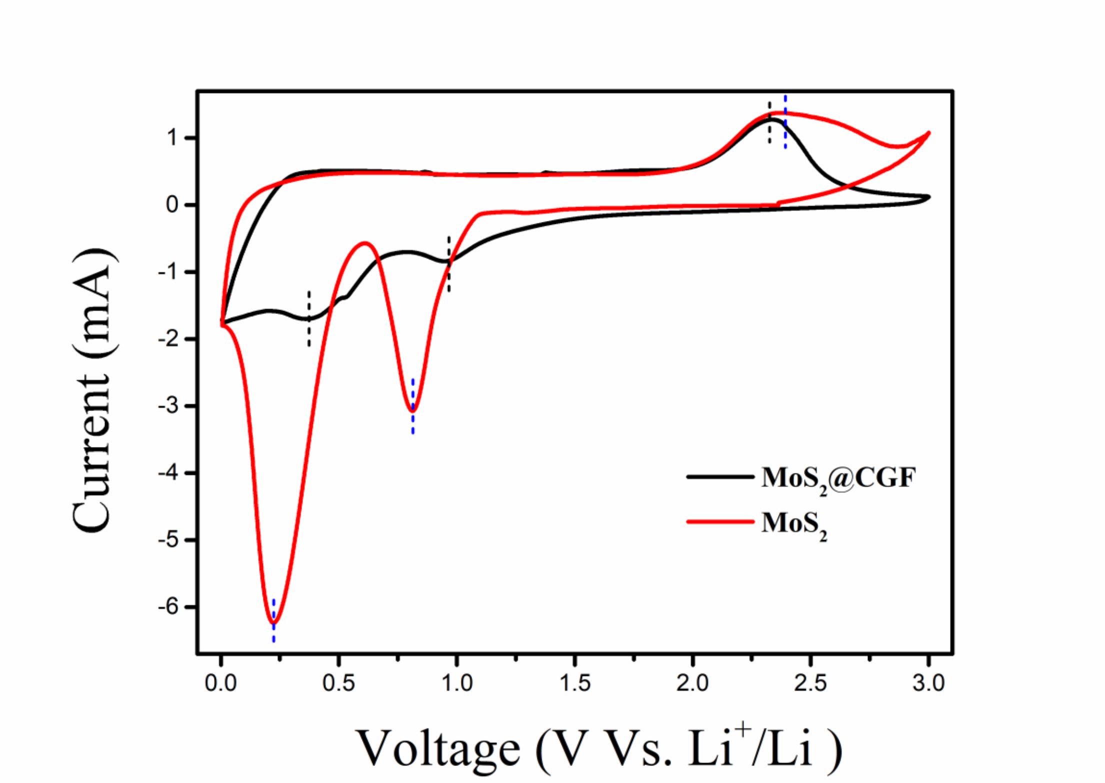

Figure S9. CV curves of MoS_2 and MoS_2@CGF electrode in first cycle.

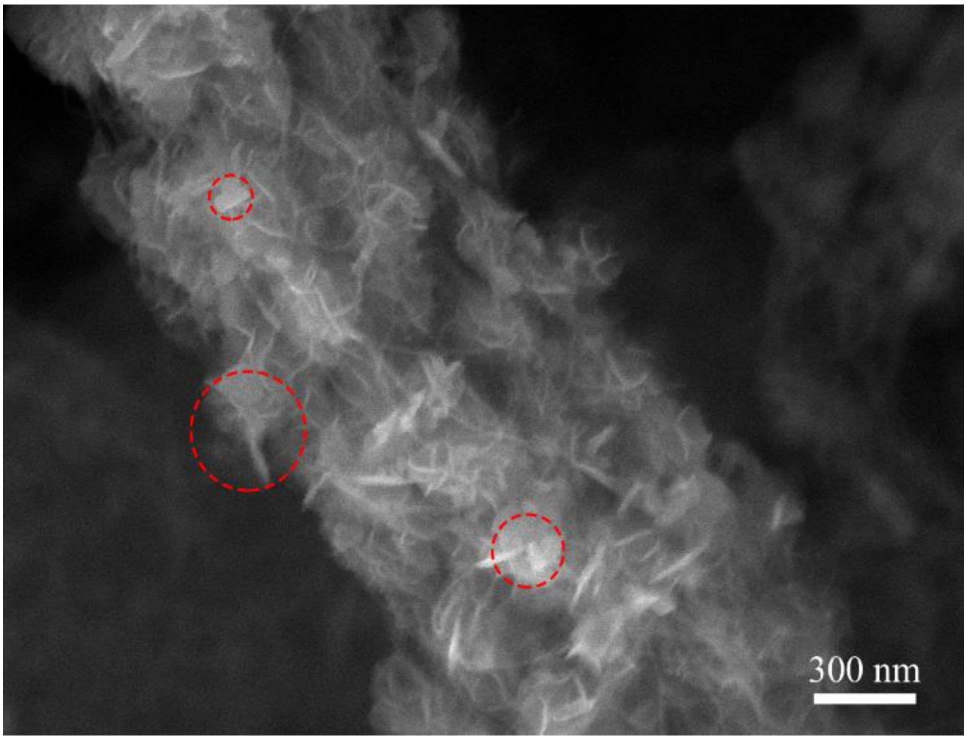

Figure S10. SEM of MoS_2@CGF with unsupported MoS_2 in the red region.

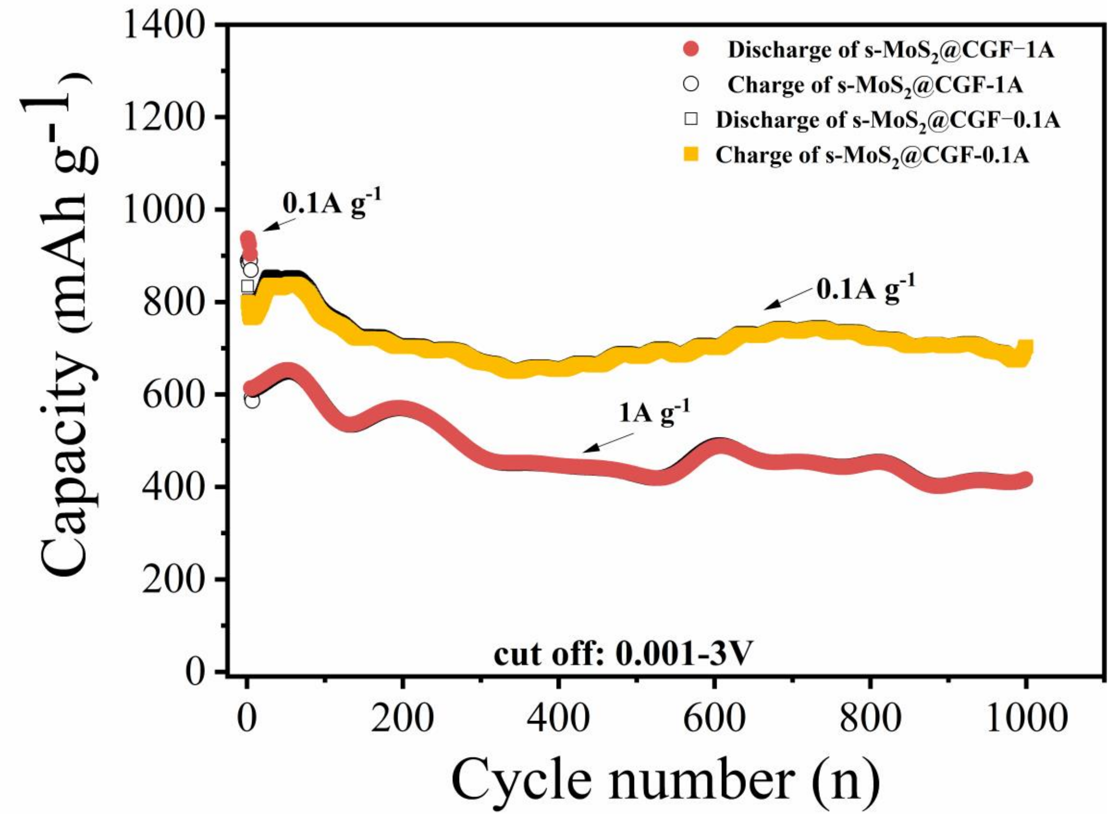

Figure S11. Cycling performance in 0.1A and 1A.

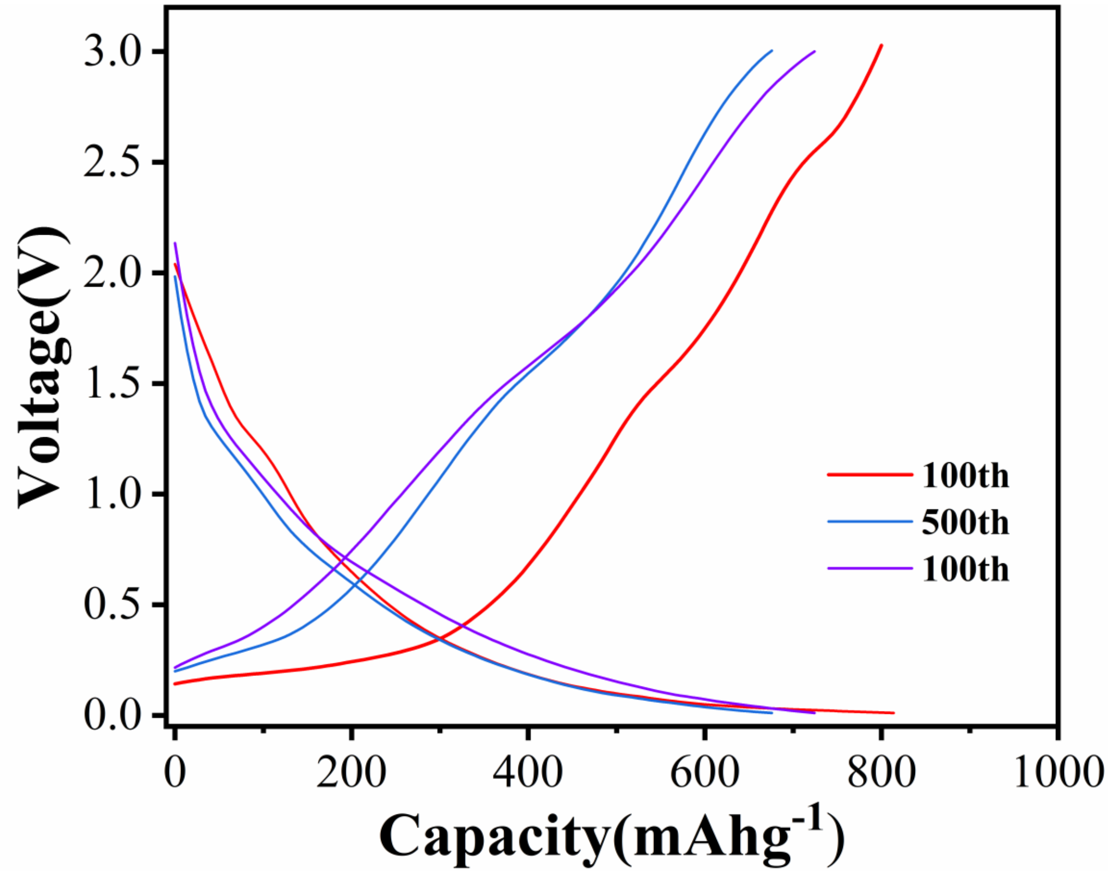

Figure S12. charge-discharge voltage profiles.

Table S1. Comparison of the electrochemical performance of MoS2/Carbon-based materials.

**Materials**	**Current density (A g^{-1})**	**Reversible capacity (mAh g^{-1})**	**Ref.**
Graphene/MoS2	0.05/5.0	735/237	[1]
MoS2/rGO	0.1/1.0	706/130	[2]
GF@CNT@MoS2-50	0.1/5.0	925/229	[3]
3DG-MoS2	0.1/2.0	949/712	[4]
N-GRs/MoS2	0.1/2.0	1000/555	[5]
Carbon nanotubes/MoS2	0.5/10.0	1089/684	[6]
MoS2-carbon porous nanorods	0.1/5.0	870/477	[7]
s-MoS_2@CGF	0.1/1.0	969/610	This work

References

1. Wang, J.; Liu, J.; Chao, D.; Yan, J.; Lin, J.; Shen, Z. X., Self-assembly of honeycomb-like MoS2 nanoarchitectures anchored into graphene foam for enhanced lithium-ion storage. *Adv Mater* **2014,** 26, (42), 7162-9.
2. Chao, Y.; Wang, K.; Jalili, R.; Morlando, A.; Qin, C.; Vijayakumar, A.; Wang, C.; Wallace, G. G., Scalable Solution Processing MoS(2) Powders with Liquid Crystalline Graphene Oxide for Flexible Freestanding Films with High Areal Lithium Storage Capacity. *ACS Appl Mater Interfaces* **2019,** 11, (50), 46746-46755.
3. Ren, J.; Ren, R.-P.; Lv, Y.-K., A flexible 3D graphene@CNT@MoS2 hybrid foam anode for high-performance lithium-ion battery. *Chemical Engineering Journal* **2018,** 353, 419-424.
4. Liu, Y.; Zhao, Y.; Jiao, L.; Chen, J., A graphene-like MoS2/graphene nanocomposite as a highperformance anode for lithium ion batteries. *J. Mater. Chem. A* **2014,** 2, (32), 13109-13115.
5. Xiao, Z.; Sheng, L.; Jiang, L.; Zhao, Y.; Jiang, M.; Zhang, X.; Zhang, M.; Shi, J.; Lin, Y.; Fan, Z., Nitrogen-doped graphene ribbons/MoS2 with ultrafast electron and ion transport for high-rate Li-ion batteries. *Chemical Engineering Journal* **2021,** 408, 127269.
6. Chen, Y. M.; Yu, X. Y.; Li, Z.; Paik, U.; Lou, X. W., Hierarchical MoS2 tubular structures internally wired by carbon nanotubes as a highly stable anode material for lithium-ion batteries. *Science Advances* 2, (7), e1600021.
7. Li, Z.; Ottmann, A.; Sun, Q.; Kast, A. K.; Wang, K.; Zhang, T.; Meyer, H.-P.; Backes, C.; Kübel, C.; Schröder, R. R.; Xiang, J.; Vaynzof, Y.; Klingeler, R., Hierarchical MoS2–carbon porous nanorods towards atomic interfacial engineering for high-performance lithium storage. *Journal of Materials Chemistry A* **2019,** 7, (13), 7553-7564.
